# Relationship between the Appearance of Symptoms and Hospital Visits in Childhood Based on Japanese Statistical Data

**DOI:** 10.3390/pediatric13040072

**Published:** 2021-11-01

**Authors:** Shiho Motoi, Akira Komatsuzaki, Sachie Ono, Hitomi Kikuchi, Asami Iguchi, Mio Susuga, Takeshi Kamoda

**Affiliations:** 1 Department of Dental Hygiene, College at Niigata, The Nippon Dental University, 1-8 Hamaura-cho, Chuo-ku, Niigata 951-8580, Japan; hsjc@ngt.ndu.ac.jp (S.M.); hitomi@ngt.ndu.ac.jp (H.K.); mio@ngt.ndu.ac.jp (M.S.); 2 Department of Preventive and Community Dentistry, School of Life Dentistry at Niigata, The Nippon Dental University, 1-8 Hamaura-cho, Chuo-ku, Niigata 951-8580, Japan; sachie@ngt.ndu.ac.jp (S.O.); kamoda-t@ngt.ndu.ac.jp (T.K.); 3 Department of Dental Anesthesiology, School of Life Dentistry at Niigata, The Nippon Dental University, Chuo-ku, Niigata 951-8580, Japan; asami@ngt.ndu.ac.jp

**Keywords:** health guidance, symptoms, disease name, health status

## Abstract

Background: Childhood health problems affect healthy growth. This study aimed to assess the symptoms and diseases requiring hospital visits commonly found in children in Japan and analyze their effects on health status. Methods: Anonymized data on 1315 children aged 6–14 years were obtained from a national survey questionnaire. The survey items addressed symptoms, disease names, and hospital visits. Associations between symptoms and other factors were examined by means of a contingency table analysis and logistic regression. Results: The proportions of responses for health status were compared for each question item; significant differences were found in age group (*p* < 0.01), subjective symptoms (*p* < 0.01), hospital visits (*p* < 0.01), and lifestyle (*p* < 0.01). The proportion of responses indicating “poor” perceived health status was high among those with subjective symptoms (4.8%) and hospital visits (4.7%). From the logistic regression, significant odds ratios were found for subjective symptoms (2.10, 95% confidence interval (C.I.) 1.15–3.83) and age group (1.98, 95% C.I. 1.05–3.72). Conclusion: Among measures to improve quality of life from childhood, comprehensive health guidance that emphasizes understanding symptoms and includes age and living conditions is important.

## 1. Introduction

There is a tendency for diseases to develop during childhood that are characteristic of the growth period [1]. These diseases are believed to be the result of diverse biological reactions and include symptoms that can readily appear because the body is morphologically and functionally immature, and allergic symptoms have a recognized tendency to increase temporarily during the growth period [2].

In dentistry, symptoms are found that develop more readily during the primary dentition period. In many countries, to encourage healthy growth and development, dental examinations are carried out at shorter intervals in children than in adults with the aim of early detection and prevention of oral disability factors. Many countries provide medical and healthcare systems that depend on the childhood stage, and the childhood stage up to puberty in particular is often the responsibility of doctors and dentists specializing in pediatrics [3]. Oral health is important for overall health; thus, it is highly necessary to further improve the plan for children’s oral health programs.

Young children often cannot be given the same medical examinations as adults because they are unable to follow the instructions or because exposure to radiation must be avoided [4]; thus, for pediatricians, any information about symptoms becomes valuable data for diagnosis.

Many countries collect data on the proportions of persons reporting particular symptoms and visiting hospitals through national statistics [5]. Japan obtains these data through the Comprehensive Survey of Living Conditions. This survey includes children, and the results are used in formulating healthcare measures. In Japan, the national government aims to improve the quality of life (QOL) throughout people’s lives from childhood by taking the lead in promoting policies such as “Healthy Parents and Children 21” [6].

In this study, data from Japanese national statistics on symptoms and diseases requiring hospital visits during childhood were used to analyze their effects on health status.

## 2. Materials and Methods

In accordance with Japanese statistics legislation, and with the permission of the Japanese Ministry of Health, Labor, and Welfare, an anonymized data file of household and health survey results for 16,262 people was obtained from the 2013 Comprehensive Survey of Living Conditions.

This study used data collected in accordance with Japanese national law in compliance with the Declaration of Helsinki. The survey and anonymization were conducted by the Ministry of Health, Labor, and Welfare. The Ministry of Health, Labor, and Welfare also obtained consent to participate in the research, but details such as the method are not disclosed. The series of studies was conducted according to the guidelines of the Japanese epidemiological survey.

From these health survey results, data for 1315 children in the 6–14-year age group were extracted and classified into two groups, corresponding to elementary and junior high school ages in Japan (Table 1).

**Figure 1:**
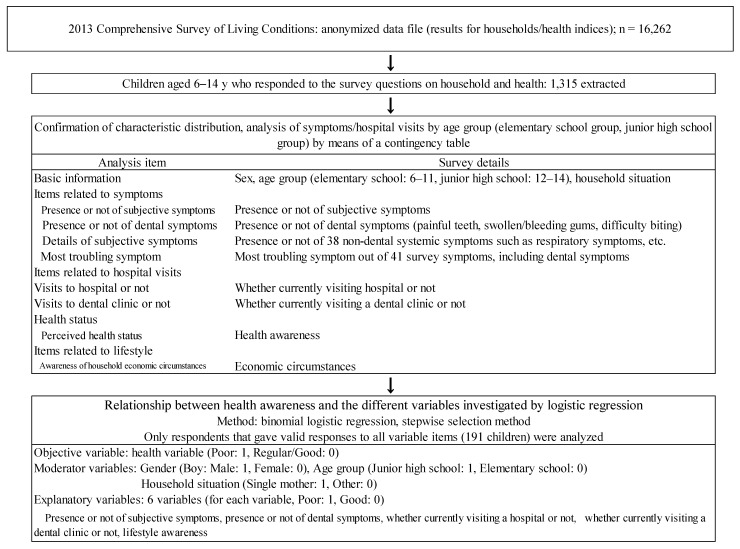
Flowchart of the data analysis in the present study.

A cross-sectional study design was adopted, and data from a single fiscal year were researched. The analysis was conducted according to the protocol shown in the flowchart in Figure 1. A comparative analysis of items was carried out using a contingency table, and the proportions of responses for items such as subjective symptoms, visits to a hospital, dental symptoms, visits to a dentist, living conditions, and perceived health status were compared. Responses for living conditions and health status were coded as either “poor” or “regular/good” for analysis. The analysis of the contingency table used the Χ^2^ test and Fisher’s exact test.

In addition, to examine symptoms and diseases requiring hospital visits common in the elementary and junior high school groups, the 41 symptoms and 40 diseases requiring hospital visits that were surveyed in the Comprehensive Survey of Living Conditions were ranked based on the proportion of responses with subjective symptoms and then compared using Spearman’s rank-order correlation coefficient.

To investigate whether symptoms or hospital visits affect perceived health status, a binomial logistic regression (stepwise selection method) was performed with perceived health status as the objective variable; then, the odds ratios were determined and compared.

For aggregate analysis, Microsoft Excel 2010 (Microsoft Japan Ltd., Tokyo, Japan) and Bell Curve for Excel (Social Survey Research Information Co., Ltd., Tokyo, Japan) were used, and the X^2^ test was conducted to determine significant differences between proportions of responses. Values of *p* < 0.05 were considered significant.

This study was approved by the ethics committee of Nippon Dental University (approval no. ECNG-R-398).

## 3. Results

### 3.1. Characteristics of the Participants

There was no sex difference in the number of subjects. The participants were divided by age into elementary and junior high school groups. There were more children in the elementary school group, but no significant difference was found in the sex ratio between the two groups.

### 3.2. Comparison of Proportions of Responses for Survey Items by Characteristic

The responses to each question are shown by group in Table 2. The items for which significant differences between groups were found were presence of subjective symptoms (*p* < 0.05) and perceived health status (*p* < 0.05). In both groups, the proportions of people with subjective symptoms and hospital visits were both <20%. The proportion of subjects responding that they had dental symptoms was low (<2%), but the proportion visiting a dental clinic was higher (6.2%).

In addition, single-mother households accounted for <10% in both groups, but the proportion of responses stating economic hardship was high, at about 70%.

### 3.3. Ranking of Symptoms and Diseases Requiring Hospital Visits

Symptoms were ranked based on the proportion of responses. The top five ranked symptoms by age group are shown in Table 3. The highest ranked symptom was blocked/runny nose in both groups. The elementary school group showed a tendency toward a higher ranking for allergic symptoms, whereas the junior high school group showed a tendency toward a higher ranking for musculoskeletal symptoms such as bone fracture or headache. The Spearman’s rank-order correlation coefficient for symptoms between the elementary and junior high school groups was 0.92 (*p* < 0.01).

Diseases requiring hospital visits are shown in Table 4. Dental diseases ranked highest in elementary school children and second highest in junior high school students; however, dental symptoms were not among the top five ranked subjective symptoms. Allergic diseases also ranked highly in both groups. The Spearman’s rank-order correlation coefficient for diseases requiring hospital visits between the elementary and junior high school groups was 0.81 (*p* < 0.01).

### 3.4. Relationships with Health Status

Table 5 shows the results of the comparison of the proportions of responses for health status by response to each question. Significant differences were found for age group (*p* < 0.01), subjective symptoms (*p* < 0.01), hospital visits (*p* < 0.01), and lifestyle awareness (*p* < 0.01). The proportion of responses indicating “poor” perceived health status was high in those with subjective symptoms (4.8%) and hospital visits (4.7%).

Table 6 shows the results of the logistic regression with health status as the objective variable. Sex, age group, and economic lifestyle awareness were inserted as moderator variables. The variables selected by the stepwise selection method were subjective symptoms, age group, lifestyle awareness, and dental clinic visits, and the variables with significant odds ratios were subjective symptoms (2.10, 95% confidence interval (C.I.) 1.15–3.83) and age group (1.98, 95% C.I. 1.05–3.72).

## 4. Discussion

The results of this study indicate that allergic diseases and physical trauma are common in childhood in Japan, and this is consistent with the results reported by Chang et al. [7] and Naranje et al. [8]. Countries other than Japan also gather data on subjective symptoms and diseases requiring hospital visits during childhood through national statistics [9], and the results suggest that some diseases occur more often during childhood.

Japan is among the countries with the longest life expectancy in the world. However, the perceived health status ranks among the lower end in the world according to the Organization for Economic Cooperation and Development [10]. The Health Promoting Schools framework activity of the World Health Organization has been developed as a Sustainable Development Goal outline aiming for sustainable societies in the future [11], and health checkups and guidance are also being implemented in all schools in Japan [12].

Moreover, according to international standards, Japan has had a high level of pediatric health and medical resources since the 1960s, and nationwide, there are over 80 pediatricians per 100,000 children [13]. This means that early-stage treatment and disease prevention can be carried out from early childhood, thereby mitigating disease progression, which is likely a factor leading to the decrease in cases showing severe symptoms. Morbidity among school-age children is decreasing, and the government’s school health statistics show a rapid reduction in infectious and dental diseases [14,15]. In addition, inflammatory symptoms and dental symptoms were not among the highest ranked diseases in this study.

One point that should be noted is that, whereas the proportion of responses for dental symptoms had a low ranking, visits to dental clinics ranked highly in both groups. Though this may appear to be a statistical abnormality, other national surveys [15] also showed that it is common for children to attend clinics on a regular basis for preventive treatments such as topical fluoride application, because abundant dental treatment resources are available for children, and many pediatric dental specialists have a private practice [16]. This is likely to have contributed to the present results.

Surveys resembling the Japanese national statistics used in this study include the Organization for Economic Cooperation and Development Health Statistics [17] and the 2011–2012 National Survey of Children’s Health [18] carried out by the United States government. Surveys other than national statistics include a study of myopia among children in Taiwan by Shao-En Chan et al. [19]. As the respective survey methodologies differ, it is not possible to make simple comparisons, but the results of these surveys appear to indicate that children have a characteristic tendency to develop allergic diseases, similar to that seen in Europe and the United States [7]. We also found a similar trend in a prior study [20]. However, the cross-sectional design of the present study prohibits any conjecture on the specific reasons for this basis for childhood specificity.

In addition, an important problem with the present study is the method of the Comprehensive Survey of Living Conditions itself. As this survey uses the same questionnaire for all age groups, items such as stiff shoulders or frequent urination are included in the options for children’s symptoms, and the options for children’s diseases include items such as osteoporosis. Therefore, the symptoms characteristic of childhood may not be covered by the available options, and many unclear responses or non-responses (elementary school group, 2.8%; junior high school group, 4.6%) were seen. The results of the logistic regression analysis showed that subjective symptoms affect perceived health status; therefore, a survey method that uses options for symptoms and diseases more appropriate for childhood is needed.

In Japan, maternal and child health issues such as child abuse and an increased prevalence of lifestyle diseases among children have become social issues of great concern [21,22]. However, no relationship between household situation and perceived health status was found in the present study. A relationship was identified between household economic circumstances and health status, so the relationship between living conditions and QOL in children needs to be investigated. In Japan, there are numerous indicators that health status disparity is increasing [23,24,25], but few studies have conducted a comprehensive analysis of the disparity during childhood that has included household situation and lifestyle habits. Additional research is needed in this area.

## 5. Conclusions

Comparisons of the proportions of responses for perceived health status by means of a contingency table showed significant differences for items such as age group, subjective symptoms, hospital visits, and lifestyle awareness. The proportion of responses indicating “poor” health status was high among those with subjective symptoms and hospital visits. Significant differences in the order of the proportion of responses were found for symptoms and diseases requiring hospital visits, but there was a trend for dental clinic visits to have a higher ranking than dental symptoms.

The results of the present study indicate that perceived health status is related to symptoms and hospital visits, and from this, it appears that there is a need to emphasize understanding of the subjective symptoms reported by children and to implement comprehensive health guidance. To improve the future outlook, a predictable pediatric healthcare system is needed.

## Figures and Tables

**Table 1:** Number of participants in each group by gender.

Subject	Male	(%)	Female	(%)	Total	(%)
Elementary school group (age 6–11 y)	445	(51.8)	414	(48.2)	859	(100.0)
Junior high school group (age 12–14 y)	239	(52.4)	217	(47.6)	456	(100.0)
Total	684	(52.0)	631	(48.0)	1315	(100.0)
					Number (%)	

**Table 2:** Proportions of responses by characteristics.

	Elementary School Group	(%)	Junior High School Group	(%)	χ2 Test
Household situation					
Single mother	56	(6.5)	43	(9.4)	
Other	802	(93.4)	413	(90.6)	
Subjective symptoms					
Yes	128	(14.9)	89	(19.5)	*
No	716	(83.4)	356	(78.1)	
Dental symptoms					
Yes	16	(1.8)	5	(1.1)	-
Hospital visits					
Yes	133	(15.5)	65	(14.3)	
No	710	(82.7)	381	(83.6)	
Dental clinic visits					
Yes	53	(6.2)	12	(2.6)	-
Health status					
Poor	4	(0.5)	812	(99.5)	*
Regular/good	9	(2.0)	433	(98.0)	
Economic circumstances					
Poverty	586	(68.2)	322	(70.6)	
Regular/comfortable	273	(31.8)	134	(29.4)	
Total ^a^	859	(100.0)	456	(100.0)	

^a^ Unclear responses excluded. * *p* < 0.05.

**Table 3:** Top five highest ranked symptoms based on proportion of responses by age group.

Rank	First	Second	Third	Fourth	Fifth
Elementary school group	Blocked/runny nose	Itching	Cough, phlegm	Fracture/sprain/dislocation	Rash
Number (%)	86 (67.2)	32 (25.0)	31 (24.2)	26 (20.3)	24 (18.8)
Junior high school group	Blocked/runny nose	Fracture/sprain/dislocation	Cough, phlegm	Itching	Tiredness, headache (same number)
Number (%)	27 (30.3)	15 (16.9)	13 (14.6)	12 (13.5)	10 (11.2)

**Table 4:** Top five highest ranked diseases requiring hospital visits by age group.

	First	Second	Third	Fourth	Fifth
Elementary school group	Dental disease	Allergic rhinitis	Atopic dermatitis	Eye disease	Asthma
Number (%)	53 (39.8)	22 (16.5)	21 (15.8)	14 (10.5)	12 (9.0)
Junior high school group	Allergic rhinitis	Dental disease	Injury/burn	Atopic dermatitis	Fracture/other skin disease (same number)
Number (%)	22 (33.8)	12 (18.5)	10 (15.4)	9 (13.8)	6 (9.2)

**Table 5:** Perceived health status by response category.

	Poor	(%)	Regular	(%)	Good	(%)	Total	χ^2^ Test
Age group								
Elementary school group	4	(0.5)	204	(25.0)	608	(74.5)	816	**
Junior high school group	9	(2.0)	171	(38.7)	262	(59.3)	442	
Sex								
Male	9	(1.4)	195	(29.7)	453	(69.0)	657	
Female	4	(0.7)	180	(30.0)	417	(69.4)	601	
Household situation								
Single mother	2	(2.1)	32	(33.7)	61	(64.2)	95	
Other	11	(1.0)	343	(29.5)	809	(70.0)	1163	
Subjective symptoms								
Yes	10	(4.8)	95	(45.5)	104	(49.8)	209	**
No	3	(0.3)	280	(26.7)	766	(73.0)	1049	
Dental symptoms								
Yes	1	(6.7)	6	(40.0)	8	(53.3)	15	
Hospital visits								
Yes	9	(4.7)	78	(40.8)	104	(54.5)	191	**
No	4	(0.4)	297	(27.8)	766	(71.8)	1067	
Dental clinic visits								
Yes	1	(2.0)	24	(47.1)	26	(50.9)	51	
Economic circumstances								
poverty	9	(1.0)	294	(33.8)	568	(65.2)	871	**
Regular/comfortable	4	(1.0)	81	(20.9)	302	(78.0)	387	

** *p* < 0.01.

**Table 6:** Results of logistic regression with perceived health status as the objective variable.

		Significance Test of Partial Regression Coefficients				95% C.I.	
Selected Explanatory Variables	Partial Regression Coefficients	Wald	*p*-Value	Determination	Odds Ratio	Lower Limit	Upper Limit
Subjective symptoms: Yes	0.74	5.87	0.02	*	2.10	1.15	3.83
Age group: Junior high school	0.65	4.53	0.03	*	1.98	1.05	3.72
Economic circumstances: Poverty	0.47	2.13	0.14		1.60	0.85	2.99
Dental clinic visits: Yes	0.46	1.78	0.18		1.59	0.80	3.15

NB: Only explanatory variables selected by stepwise selection method are shown. * *p* < 0.05. *n* = 191; coefficient of determination R^2^ = 0.05; percentage of correct classifications, 60.7%.

## Data Availability

Not applicable.
